# Supplementary material for: Incoherent merger network for robust ratiometric gene expression response
Source: Nucleic Acids Res. 2023 Feb 25;51(6):2963–73. doi: 10.1093/nar/gkad087 (PMC10085686; doi:10.1093/nar/gkad087)
Supplement: gkad087_Supplemental_File [file gkad087_supplemental_file.pdf]

Incoherent merger network for robust ratiometric gene expression response

Supplementary Information

Ukjin Kwon[1], Hsin-ho Huang[2], Jorge L. Chávez[3], Kathryn Beabout[3,4], Svetlana Harbaugh[3], Domitilla Del Vecchio[2,5,*]

[1] Department of Electrical Engineering and Computer Science, Massachusetts Institute of Technology, Cambridge, MA, USA.
[2] Department of Mechanical Engineering, Massachusetts Institute of Technology, Cambridge, MA, USA.
[3] 711th Human Performance Wing, Air Force Research Laboratory, Wright Patterson Air Force Base, OH, USA.
[4] UES, Inc., Dayton, OH, USA.
[5] Synthetic Biology Center, Massachusetts Institute of Technology, Cambridge, MA, USA.

[*] Corresponding author.

Supplementary Note 1 Modeling framework

In Supplementary Note 1.1, we first derive the steady state value of the output protein P_Y of the merger network motif in Figure 2a. Then we also derive the steady state value of the output protein P_Y of the ratiometric sensor in Figure 3a. For both cases, we prove that the steady state value of the output protein P_Y is, with certain conditions satisfied, proportional to the ratio of the two inputs, Y/X, with quantitatively tunable sensitivity, and it is robust to the resource competition. In Supplementary Note 1.2, we derive the steady state value of the output protein P_Y of the control circuit of the ratiometric sensor (Figure 4a). With the same conditions that Figure 3a works as the ratiometric sensor, the steady state value of P_Y of Figure 4a is no longer proportional to the ratio of the two inputs, Y/X, and it is function of the resource, which implies it is no longer robust to the resource competition. In Supplementary Note 1.3, we derive the steady state value of the output protein P_Y of Figure 5a - incoherent merger network and Figure 5a - broken merging, which has an additional resource competitor module of Figure 3a and Figure 4a, respectively. We prove that the steady state value of the output protein P_Y of Figure 5a - incoherent merger network is the same as that of Figure 3a, which shows that our ratiometric sensor is robust to the resource competition. In addition, the steady state value of the output protein P_Y of Figure 5a - broken merging is smaller than that of Figure 4a, which implies the control circuit is not robust to the resource competition.

Supplementary Note 1.1 The steady state value of the output protein P_Y of the merger network motif in Figure 2a and the ratiometric sensor in Figure 3a

In this section, we first derive the steady state value of the output protein P_Y of Figure 2a as functions of two inputs X and Y. Assuming that the production of P_X and of P_Y can be written as one-step enzymatic reactions, we have:

$$\mathrm{X} + \mathrm{R} \xrightarrow{k_X} \mathrm{P}_X + \mathrm{X} + \mathrm{R}, \quad \mathrm{Y} + \mathrm{R} \xrightarrow{k_Y} \mathrm{P}_Y + \mathrm{Y} + \mathrm{R}, \tag{1}$$

where k_X and k_Y are production rates constants. The interaction $P_X \dashv P_Y$ in Figure 2a can be written as follows:

$$\mathrm{P}_X + \mathrm{P}_Y \underset{b}{\overset{a}{\rightleftharpoons}} \mathrm{C} \xrightarrow{k} \mathrm{P}_X. \tag{2}$$

We also consider dilution, $\mathrm{P}_X, \mathrm{P}_Y, C \xrightarrow{\gamma} \emptyset$, where γ is the dilution rate constant. By assuming the complex concentration C at the quasi-steady state and by letting $P_{XT} = P_X + C$ be the total concentration of P_X, we obtain the reduced ODE model:

$$\begin{aligned} \frac{d}{dt}P_{XT} &= k_X XR - \gamma P_{XT}, \\ \frac{d}{dt}P_Y &= k_Y YR - (k+\gamma)\frac{P_Y P_{XT}}{K + P_Y} - \gamma P_Y, \end{aligned} \tag{3}$$

where $K = (b + k + \gamma)/a$ is the Michaelis-Menten constant of the protease reaction (2). To achieve robust ratiometric sensing, we require that (A1) $P_Y \ll K$ and (A2) $\gamma \ll P_{XT}(k+\gamma)/K$. With these assumptions, we obtain that the steady state value of the output protein P_Y is given by

$$P_Y = \frac{\gamma k_Y K}{(k+\gamma)k_X} \cdot \frac{Y}{X} = c \cdot \left(\frac{Y}{X}\right), \quad c := \frac{\gamma k_Y K}{(k+\gamma)k_X}. \tag{4}$$

Therefore, the steady state value of the output protein P_Y is proportional to the ratio Y/X and this ratio is independent of the resource level R.

Next, we derive the steady state value of the output protein P_Y of Figure 3a as functions of two inputs X and Y. Letting R_X be the transcriptional repressor, D_{RX} be the DNA where the gene of R_X is encoded, m_{RX}

be the mRNA of R_X, and R be the ribosome, the reactions modeling the production of R_X can be written as follows:

$$\mathrm{D}_{RX} \xrightarrow{d_{RX}} \mathrm{m}_{RX} + \mathrm{D}_{RX}, \quad \mathrm{m}_{RX} + \mathrm{R} \underset{b_{RX}}{\overset{a_{RX}}{\rightleftharpoons}} \mathrm{C}_{RX} \xrightarrow{k_{RX}} \mathrm{R}_X + \mathrm{m}_{RX} + \mathrm{R}, \quad R_X, m_{RX}, C_{RX} \xrightarrow{\gamma} \emptyset, \tag{5}$$

where C_{RX} is a complex, a_{RX} and b_{RX} are binding and unbinding rate constants, respectively, d_{RX} is the mRNA production rate constant, k_{RX} is the protein production rate constant, and γ is the dilution rate constant. The first reaction models mRNA production, the second reaction models translation, and the third reaction captures dilution. Letting D_X be the DNA where the gene of P_X is encoded, and m_X be the mRNA of P_X, the reactions modeling the production of P_X can be written as follows:

$$\begin{aligned} &\mathrm{R}_X + \mathrm{D}_X \underset{b_1}{\overset{a_1}{\rightleftharpoons}} \mathrm{C}_1, \quad \mathrm{X} + \mathrm{R}_X \underset{b_2}{\overset{a_2}{\rightleftharpoons}} \mathrm{C}_2, \quad \mathrm{D}_X \xrightarrow{k_1} \mathrm{D}_X + \mathrm{m}_X, \\ &\mathrm{m}_X + \mathrm{R} \underset{b_3}{\overset{a_3}{\rightleftharpoons}} \mathrm{C}_3 \xrightarrow{k_3} \mathrm{P}_X + \mathrm{m}_X + \mathrm{R}, \quad \mathrm{P}_X, \mathrm{m}_X, \mathrm{C}_i \xrightarrow{\gamma} \emptyset, i \in \{1,2,3\}, \end{aligned} \tag{6}$$

where C_i ($i = 1, 2, 3$) are complexes, a_i and b_i ($i = 1, 2, 3$) are binding and unbinding rate constants, respectively, k_1 is the mRNA production rate constant, k_3 is the protein production rate constant, and γ is the dilution rate constant. The first reaction models that R_X represses transcription by sequestering D_X into a transcriptionally inactive complex C_1, the second reaction models that inducer X sequesters the repressor so it is unable to bind DNA, the third reaction models mRNA production, the fourth reaction models translation, and the fifth reaction reflects dilution.

Similarly, letting A_Y be the transcriptional activator, D_{AY} be the DNA where the gene of A_Y is encoded, and m_{AY} be the mRNA of A_Y, the reactions modeling the production of A_Y can be written as follows:

$$\mathrm{D}_{AY} \xrightarrow{d_{AY}} \mathrm{m}_{AY} + \mathrm{D}_{AY}, \quad \mathrm{m}_{AY} + \mathrm{R} \underset{b_{AY}}{\overset{a_{AY}}{\rightleftharpoons}} \mathrm{C}_{AY} \xrightarrow{k_{AY}} \mathrm{A}_Y + \mathrm{m}_{AY} + \mathrm{R}, \quad \mathrm{A}_Y, \mathrm{m}_{AY}, \mathrm{C}_{AY} \xrightarrow{\gamma} \emptyset, \tag{7}$$

where C_{AY} is a complex, a_{AY} and b_{AY} are binding and unbinding rate constants, respectively, d_{AY} is the mRNA production rate constant, k_{AY} is the protein production rate constant. The first reaction models mRNA production, the second reaction models translation, and the third reaction captures dilution. Letting D_Y be the DNA where the gene of P_Y is encoded, and m_Y be the mRNA of P_Y, the reactions modeling the production of P_Y can be written as follows:

$$\begin{aligned} &\mathrm{Y} + \mathrm{A}_Y \underset{b_4}{\overset{a_4}{\rightleftharpoons}} \mathrm{C}_4, \quad \mathrm{D}_Y + \mathrm{C}_4 \underset{b_5}{\overset{a_5}{\rightleftharpoons}} \mathrm{C}_5 \xrightarrow{k_5} \mathrm{m}_Y + \mathrm{D}_Y + \mathrm{C}_4, \\ &\mathrm{m}_Y + \mathrm{R} \underset{b_6}{\overset{a_6}{\rightleftharpoons}} \mathrm{C}_6 \xrightarrow{k_6} \mathrm{P}_Y + \mathrm{m}_Y + \mathrm{R}, \quad \mathrm{P}_Y, \mathrm{m}_Y, \mathrm{C}_i \xrightarrow{\gamma} \emptyset, \end{aligned} \tag{8}$$

where C_i ($i = 4, 5, 6$) are complexes, a_i and b_i ($i = 4, 5, 6$) are binding and unbinding rate constants, respectively, k_5 is the mRNA production rate constant, k_6 is the protein production rate constant. The first reaction models that inducer Y combines with the activator so it is able to bind DNA, the second reaction models mRNA production, the third reaction models translation, and the fourth reaction reflects dilution.

The interaction $P_X \dashv P_Y$ can be implemented by having P_X enhance the degradation of P_Y, and take P_X as a protease, which binds to its target on protein P_Y, forming a complex C, which leads to the release of P_X and degradation of P_Y:

$$\mathrm{P}_X + \mathrm{P}_Y \underset{b}{\overset{a}{\rightleftharpoons}} \mathrm{C} \xrightarrow{k} \mathrm{P}_X. \tag{9}$$

By combining reactions in (5) - (9), we obtain the following ODE model:

$$\frac{d}{dt} C_{RX} = a_{RX} m_{RX} R - (b_{RX} + k_{RX} + \gamma) C_{RX}, \tag{10a}$$

$$\frac{d}{dt}m_{RX} = d_{RX}D_{RX} - a_{RX}m_{RX}R + (b_{RX} + k_{RX})C_{RX} - \gamma m_{RX}, \tag{10b}$$

$$\frac{d}{dt}R_X = k_{RX}C_{RX} - a_1 R_X D_X + b_1 C_1 - a_2 X R_X + b_2 C_2 - \gamma R_X, \tag{10c}$$

$$\frac{d}{dt}C_1 = a_1 R_X D_X - (b_1 + \gamma)C_1, \tag{10d}$$

$$\frac{d}{dt}C_2 = a_2 X R_X - (b_2 + \gamma)C_2, \tag{10e}$$

$$\frac{d}{dt}C_3 = a_3 m_X R - (b_3 + k_3 + \gamma)C_3, \tag{10f}$$

$$\frac{d}{dt}m_X = k_1 D_X - a_3 m_X R + (b_3 + k_3)C_3 - \gamma m_X, \tag{10g}$$

$$\frac{d}{dt}P_X = k_3 C_3 - a P_X P_Y + (b + k)C - \gamma P_X, \tag{10h}$$

$$\frac{d}{dt}C_{AY} = a_{AY}m_{AY}R - (b_{AY} + k_{AY} + \gamma)C_{AY}, \tag{10i}$$

$$\frac{d}{dt}m_{AY} = d_{AY}D_{AY} - a_{AY}m_{AY}R + (b_{AY} + k_{AY})C_{AY} - \gamma m_{AY}, \tag{10j}$$

$$\frac{d}{dt}A_Y = k_{AY}C_{AY} - a_4 Y A_Y + b_4 C_4 - \gamma A_Y, \tag{10k}$$

$$\frac{d}{dt}C_4 = a_4 Y A_Y - (b_4 + \gamma)C_4 + (b_5 + k_5)C_5 - a_5 D_Y C_4, \tag{10l}$$

$$\frac{d}{dt}C_5 = a_5 D_Y C_4 - (b_5 + k_5 + \gamma)C_5, \tag{10m}$$

$$\frac{d}{dt}C_6 = a_6 m_Y R - (b_6 + k_6 + \gamma)C_6, \tag{10n}$$

$$\frac{d}{dt}m_Y = k_5 C_5 - a_6 m_Y R + (b_6 + k_6)C_6 - \gamma m_Y, \tag{10o}$$

$$\frac{d}{dt}P_Y = k_6 C_6 - a P_X P_Y + bC - \gamma P_Y, \tag{10p}$$

$$\frac{d}{dt}C = a P_X P_Y - (b + k + \gamma)C. \tag{10q}$$

We refer to (10) as the full model. From (10d) - (10f), we obtain the steady state values of the complexes as follows:

$$C_1 = \frac{R_X D_X}{K_1}, \quad C_2 = \frac{X R_X}{K_2}, \quad C_3 = \frac{m_X R}{K_3}, \tag{11}$$

where $K_1 = (b_1 + \gamma)/a_1$ and $K_2 = (b_2 + \gamma)/a_2$ are dissociation constants, and $K = (b_3 + k_3 + \gamma)/a_3$ is the Michaelis-Menten constant. With (11), (10d) - (10f), and letting $R_{XT} = R_X + C_1 + C_2$ be the total concentration of the repressor, we obtain follows:

$$R_{XT} = R_X + C_1 + C_2 = R_X(1 + \frac{D_X}{K_1} + \frac{X}{K_2}) \rightarrow R_X = \frac{R_{XT}}{1 + \frac{D_X}{K_1} + \frac{X}{K_2}} \approx \frac{R_{XT}K_2}{X} \quad (\text{if } D_X \ll K_1, K_2 \ll X). \tag{12}$$

Also, with (11) and (12), and letting $D_{XT} = D_X + C_1$ be the total concentration of the DNA, we obtain follows:

$$D_{XT} = D_X + C_1 = D_X(1 + \frac{R_{XT}K_2}{K_1 X}) \rightarrow D_X = \frac{D_{XT}}{1 + \frac{R_{XT}K_2}{K_1 X}} \approx \frac{D_{XT}K_1 X}{R_{XT}K_2} \quad (\text{if } X \ll \frac{R_{XT}K_2}{K_1}). \tag{13}$$

Also, with (11) and (12), and letting $X_T = X + C_2$ be the total concentration of the first input, we obtain follows:

$$X_T = X + C_2 = X(1 + \frac{R_{XT}}{X}) \rightarrow X = \frac{X_T}{1 + \frac{R_{XT}}{X}} \approx X_T \quad (\text{if } R_{XT} \ll X). \tag{14}$$

Therefore, from (12) - (14), we obtain the steady state value of D_X as follows:

$$D_X = \frac{K_1 D_{XT}}{K_2 R_{XT}} X_T, \tag{15}$$

which is proportional to X_T. Here, the assumptions $D_X \ll K_1, K_2 \ll X, X \ll \frac{R_{XT}K_2}{K_1}, R_{XT} \ll X$ should be satisfied, and these can be easily achieved by selecting a repressor based transcriptional sensor that has wide linear range.

From (10l) - (10n), we obtain the steady state values of the complexes as follows:

$$C_4 = \frac{Y A_Y}{K_4}, \quad C_5 = \frac{D_Y C_4}{K_5}, \quad C_6 = \frac{m_Y R}{K_6}, \tag{16}$$

where $K_4 = (b_4 + \gamma)/a_4$ is the dissociation constant, and $K_5 = (b_5 + k_5 + \gamma)/a_5$ and $K_6 = (b_6 + k_6 + \gamma)/a_6$ is the Michaelis-Menten constant. With (16) and letting $D_{YT} = D_Y + C_5$ be the total concentration of the DNA, we obtain follows:

$$D_{YT} = D_Y + C_5 = D_Y(1 + \frac{C_4}{K_5}) \rightarrow D_Y = \frac{D_{YT}}{1 + \frac{C_4}{K_5}} \approx D_{YT} \quad (\text{if } C_4 \ll K_5). \tag{17}$$

Also, with (16) and letting $Y_T = Y + C_4 + C_5$ be the total amount of the second input, and letting $A_{YT} = A_Y + C_4 + C_5$ be the total concentration of the activator, we have

$$Y_T = Y + C_4 + C_5 = Y(1 + \frac{A_Y}{K_4}(1 + \frac{D_Y}{K_5})) \rightarrow Y = \frac{Y_T}{1 + \frac{A_Y}{K_4}(1 + \frac{D_Y}{K_5})} \approx Y_T \quad (\text{if } A_Y \ll K_4, D_Y \ll K_5), \tag{18}$$

$$A_{YT} = A_Y + C_4 + C_5 = A_Y(1 + \frac{Y}{K_4}(1 + \frac{D_Y}{K_5})) \rightarrow A_Y = \frac{A_{YT}}{1 + \frac{Y_T}{K_4}(1 + \frac{D_Y}{K_5})} \approx A_{YT} \quad (\text{if } Y \ll K_4). \tag{19}$$

Therefore, from (16) - (19), we obtain the steady state value of C_5 as follows:

$$C_5 = \frac{D_{YT} A_{YT} Y_T}{K_4 K_5}, \tag{20}$$

which is proportional to Y_T. Here, the assumptions $(Y A_Y)/K_4 \ll K_5, A_Y \ll K_4, D_Y \ll K_5, Y \ll K_4$ should be satisfied, and these can be easily achieved by selecting an activator based transcriptional sensor that has wide linear range.

Letting $dC_{RX}/dt = 0$ in (10a), the steady state value of the complex, C_{RX}, can be obtained as follows:

$$C_{RX} = \frac{m_{RX} R}{K_{RX}}, \tag{21}$$

where $K_{RX} = (b_{RX} + k_{RX} + \gamma)/a_{RX}$ is the Michaelis-Menten constant. With (10a), (10b) and (21), and letting $m_{RXT} = m_{RX} + C_{RX}$ be the total concentration of the repressor mRNA, we obtain follows:

$$m_{RXT} = \frac{d_{RX}D_{RX}}{\gamma} = m_{RX} + C_{RX} = m_{RX}(1 + \frac{R}{K_{RX}}) \rightarrow C_{RX} = \frac{\frac{R}{K_{RX}}}{1 + \frac{R}{K_{RX}}} \frac{d_{RX}D_{RX}}{\gamma} \approx \frac{d_{RX}D_{RX}}{\gamma} \quad (\text{if } K_{RX} \ll R). \tag{22}$$

From (10f) - (10g), letting $m_{XT} = m_X + C_3$ be the total concentration of the mRNA, we obtain follows:

$$m_{XT} = \frac{k_1 D_X}{\gamma} = m_X + C_3 = m_X(1 + \frac{R}{K_3}) \rightarrow m_X = \frac{k_1 D_X}{\gamma(1 + \frac{R}{K_3})} \approx \frac{k_1 D_X}{\gamma} \quad (\text{if } R \ll K_3). \tag{23}$$

By combining (11), (15) and (23), we obtain follows:

$$C_3 = \frac{\gamma k_1 K_1 D_{XT}}{k_{RX} d_{RX} K_2 K_3 D_{RX}} R X_T, \tag{24}$$

which is proportional to R and X_T. Here, the assumption $K_{RX} \ll R \ll K_3$ should be satisfied and we can increase K_3 by decreasing TIR of the protease P_X in (6) and decrease K_{RX} by increasing TIR of the repressor R_X in (5) [44].

Letting $dC_{AY}/dt = 0$ in (10i), the steady state value of the complex, C_{AY}, can be obtained as follows:

$$C_{AY} = \frac{m_{AY} R}{K_{AY}}, \tag{25}$$

where $K_{AY} = (b_{AY} + k_{AY} + \gamma)/a_{AY}$ is the Michaelis-Menten constant. With (10i), (10j) and (25), and letting $m_{AYT} = m_{AY} + C_{AY}$ be the total concentration of the activator mRNA, we obtain follows:

$$m_{AYT} = \frac{d_{AY}D_{AY}}{\gamma} = m_{AY} + C_{Ay} = m_{AY}(1 + \frac{R}{K_{AY}}) \rightarrow C_{AY} = \frac{\frac{R}{K_{AY}}}{1 + \frac{R}{K_{AY}}} \frac{d_{AY}D_{AY}}{\gamma} \approx \frac{d_{AY}D_{AY}}{\gamma} \quad (\text{if } K_{AY} \ll R). \tag{26}$$

From (10n), (10o) and (16), letting $m_{YT} = m_Y + C_6$ be the total concentration of the mRNA, we obtain follows:

$$m_{YT} = \frac{k_5 C_5}{\gamma} = m_Y + C_6 = m_Y(1 + \frac{R}{K_6}) \rightarrow m_Y = \frac{k_5 C_5}{\gamma(1 + \frac{R}{K_6})} \approx \frac{k_5 C_5}{\gamma} \quad (\text{if } R \ll K_6). \tag{27}$$

By combining (16), (20) (26) and (27), we obtain follows:

$$C_6 = \frac{k_5 D_{YT} k_{AY} d_{AY} D_{AY}}{\gamma^3 K_4 K_5 K_6} R Y_T, \tag{28}$$

which is proportional to R and Y_T. Here, the assumption $K_{AY} \ll R \ll K_6$ should be satisfied and we can increase K_6 by decreasing TIR of the output protein P_Y in (8) and increase K_{AY} by decreasing TIR of the activator A_Y in (7) [44].

Letting $dC/dt = 0$ in (10q), the steady state value of the complex C can be obtained as follows:

$$C = \frac{P_X P_Y}{K}, \tag{29}$$

where $K = (b + k + \gamma)/a$ is the Michaelis-Menten constant. From (10h), (10p), (10q), (29), and by letting $P_{XT} = P_X + C$ be the total concentration of P_X, we obtain the reduced ODE model:

$$\begin{aligned} \frac{d}{dt} P_{XT} &= k_3 C_3 - \gamma P_{XT}, \\ \frac{d}{dt} P_Y &= k_6 C_6 - (k + \gamma) \frac{P_Y P_{XT}}{K + P_Y} - \gamma P_Y, \end{aligned} \tag{30}$$

where the steady state value of C_3 and C_6 are in (24), (28), respectively. Then (30) can be written as follows:

$$\begin{aligned}\frac{d}{dt}P_{XT} &= k_X X_T R - \gamma P_{XT},\\ \frac{d}{dt}P_Y &= k_Y Y_T R - (k+\gamma)\frac{P_Y P_{XT}}{K+P_Y} - \gamma P_Y,\end{aligned} \tag{31}$$

where $k_X = (\gamma k_1 k_3 K_1 D_{XT})/(k_{RX} d_{RX} K_2 K_3 D_{RX})$ and $k_Y = (k_5 k_6 D_{YT} k_{AY} d_{AY} D_{AY})/(\gamma^3 K_4 K_5 K_6)$, which is equivalent to (31) in main text. With (A1) and (A2) assumptions in the main text, when we substitute k_X and k_Y in (3) of the main text, we obtain the steady state value of the output protein P_Y as follows:

$$P_Y = \frac{d_{RX} k_{RX} d_{AY} k_{AY} k_5 k_6 K K_2 K_3 D_{YT} D_{RX} D_{AY}}{\gamma^3 (k+\gamma) k_1 k_3 K_1 K_4 K_5 K_6 D_{XT}} \cdot \frac{Y_T}{X_T} = c\cdot\Big(\frac{Y_T}{X_T}\Big), \quad c := \frac{d_{RX} k_{RX} d_{AY} k_{AY} k_5 k_6 K K_2 K_3 D_{YT} D_{RX} D_{AY}}{\gamma^3 (k+\gamma) k_1 k_3 K_1 K_4 K_5 K_6 D_{XT}}, \tag{32}$$

which is proportional to Y_T/X_T, and it is not a function of R. In addition, the slope c can also be quantitatively tuned by varying the strength of ribosome binding site (RBS) of the output protein P_Y. To be more specific, increasing (decreasing) RBS of the output protein, we can increase (decrease) a_6 in (4), which will decrease (increase) $K_6 = (b_6 + k_6 + \gamma)/a_6$, and eventually the steady state value of the output protein P_Y will increase (decrease) because it is proportional to $1/K_6$ [44]. In summary, we can build a robust and quantitatively tunable ratiometric sensor if three assumptions (A0) - (A2) are satisfied.

Supplementary Note 1.2 The steady state value of the output protein P_Y of the control circuit in Figure 4a

In this section, we derive the steady state value of the output protein P_Y of Figure 4a as functions of two inputs X and Y. In Figure 4a, P_X cannot degrade P_Y. This implies that for Figure 4a, (5), (6), (7) and (8) represent the entire reactions. Then we obtain the steady state value of the output protein P_Y of Figure 4a as $k_6 C_6/\gamma$, where C_6 is in (28), which is different from that of Figure 3a in (32), and there is a conservation law for the total concentration of the ribosome, R_{tot}, as follows:

$$R_{tot} = R + C_3 + C_6. \tag{33}$$

Here, we do not contain C_{RX} and C_{AY} in the conservation law because they are chromosomally integrated in Marionette strain and constitutively expressed, so their impact is already reflected in R_{tot}. Then, by combining (11), (16) and (33), we can obtain the steady state value of the output protein P_Y as follows (we refer to it as reduced model):

$$\begin{aligned}P_Y &= \frac{k_5 k_6 k_{AY} d_{AY} D_{YT} D_{AY} Y_T}{\gamma^4 K_4 K_5 K_6} R = \frac{k_6 R_{tot}}{\gamma} \frac{c_2 Y_T}{1 + c_1 X_T + c_2 Y_T},\\ \text{where} \quad c_1 &= \frac{\gamma k_1 K_1 D_{XT}}{k_{RX} d_{RX} K_2 K_3 D_{RX}}, \quad c_2 = \frac{k_5 k_{AY} d_{AY} D_{YT} D_{AY}}{\gamma^3 K_4 K_5 K_6},\end{aligned} \tag{34}$$

and (34) is not a ratio function.

Supplementary Note 1.3 The steady state value of the output protein P_Y of Figure 5a - incoherent merger network and Figure 5a - broken merging

Comparing to Figure 3a and Figure 4a, Figure 5a has an additional resource competitor module. Letting R_Z be the third transcriptional repressor, D_{RZ} be the third DNA where the gene of R_Z is encoded, and m_{RZ} be the mRNA of R_Z, the reactions modeling the production of R_Z can be written as follows:

$$D_{RZ} \xrightarrow{d_{RZ}} m_{RZ} + D_{RZ}, \quad m_{RZ} + R \underset{b_{RZ}}{\overset{a_{RZ}}{\rightleftharpoons}} C_{RZ} \xrightarrow{k_{RZ}} R_Z + m_{RZ} + R, \quad R_Z, m_{RZ}, C_{RZ} \xrightarrow{\gamma} \emptyset, \tag{35}$$

where C_{RZ} is a complex, a_{RZ} and b_{RZ} are binding and unbinding rate constants, respectively, d_{RZ} is the mRNA production rate constant, k_{RZ} is the protein production rate constant. The first reaction models mRNA production, the second reaction models translation, and the third reaction captures dilution. Letting D_Z be the third DNA where the gene of P_Z is encoded, and m_Z be the mRNA of P_Z, the reactions modeling the production of P_Z can be written as follows:

$$\begin{aligned}&R_Z + D_Z \underset{b_7}{\overset{a_7}{\rightleftharpoons}} C_7, \quad Z + R_Z \underset{b_8}{\overset{a_8}{\rightleftharpoons}} C_8, \quad D_Z \xrightarrow{k_7} D_Z + m_Z,\\ &m_Z + R \underset{b_9}{\overset{a_9}{\rightleftharpoons}} C_9 \xrightarrow{k_9} P_Z + m_Z + R, \quad P_Z, m_Z, C_i \xrightarrow{\gamma} \emptyset, i \in \{7,8,9\},\end{aligned} \tag{36}$$

where C_i ($i = 7,8,9$) are complexes, a_i and b_i ($i = 7,8,9$) are binding and unbinding rate constants, respectively, k_7 is the mRNA production rate constant, k_9 is the protein production rate constant. The first reaction models that R_Z represses transcription by sequestering D_Z into a transcriptionally inactive complex C_7, the second reaction models that inducer Z sequesters the repressor so it is unable to bind DNA, the third reaction models mRNA production, the fourth reaction models translation, and the fifth reaction reflects dilution.

By combining reactions in (35) - (36), we obtain the following ODE model:

$$\frac{d}{dt}C_{RZ} = a_{RZ}m_{RZ}R - (b_{RZ} + k_{RZ} + \gamma)C_{RZ}, \tag{37a}$$

$$\frac{d}{dt}m_{RZ} = d_{RZ}D_{RZ} - a_{RZ}m_{RZ}R + (b_{RZ} + k_{RZ})C_{RZ} - \gamma m_{RZ}, \tag{37b}$$

$$\frac{d}{dt}R_Z = k_{RZ}C_{RZ} - a_7R_ZD_Z + b_7C_7 - a_8ZR_Z + b_8C_8 - \gamma R_Z, \tag{37c}$$

$$\frac{d}{dt}C_7 = a_7R_ZD_Z - (b_7 + \gamma)C_7, \tag{37d}$$

$$\frac{d}{dt}C_8 = a_8ZR_Z - (b_8 + \gamma)C_8, \tag{37e}$$

$$\frac{d}{dt}C_9 = a_9m_ZR - (b_9 + k_9 + \gamma)C_9, \tag{37f}$$

$$\frac{d}{dt}m_Z = k_7D_Z - a_9m_ZR + (b_9 + k_9)C_9 - \gamma m_Z, \tag{37g}$$

$$\frac{d}{dt}P_Z = k_9C_9 - \gamma P_Z. \tag{37h}$$

From (37d) - (37f), we obtain the steady state values of the complexes as follows:

$$C_7 = \frac{R_ZD_Z}{K_7}, \quad C_8 = \frac{ZR_Z}{K_8}, \quad C_9 = \frac{m_ZR}{K_9}, \tag{38}$$

where $K_7 = (b_7 + \gamma)/a_7$ and $K_8 = (b_8 + \gamma)/a_8$ are dissociation constants, and $K_9 = (b_9 + k_9 + \gamma)/a_9$ is the Michaelis-Menten constant. With (38), (37d) - (37f), and letting $R_{ZT} = R_Z + C_7 + C_8$ be the total concentration of the third repressor, we obtain follows:

$$R_{ZT} = R_Z + C_7 + C_8 = R_Z(1 + \frac{D_Z}{K_7} + \frac{Z}{K_8}) \rightarrow R_Z = \frac{R_{ZT}}{1 + \frac{D_Z}{K_7} + \frac{Z}{K_8}} \approx \frac{R_{ZT}K_8}{Z} \quad (\text{if } D_Z \ll K_7, K_8 \ll Z). \tag{39}$$

Also, with (38) and (39), and letting $D_{ZT} = D_Z + C_7$ be the total concentration of the third DNA, we obtain follows:

$$D_{ZT} = D_Z + C_7 = D_Z(1 + \frac{R_{ZT}K_8}{K_7Z}) \rightarrow D_Z = \frac{D_{ZT}}{1 + \frac{R_{ZT}K_8}{K_7Z}} \approx \frac{D_{ZT}K_7Z}{R_{ZT}K_8} \quad (\text{if } Z \ll \frac{R_{ZT}K_8}{K_7}). \tag{40}$$

Also, with (38) and (39), and letting $Z_T = Z + C_8$ be the total concentration of the third input, we obtain follows:

$$Z_T = Z + C_8 = Z(1 + \frac{R_{ZT}}{Z}) \rightarrow Z = \frac{Z_T}{1 + \frac{R_{ZT}}{Z}} \approx Z_T \quad (\text{if } R_{ZT} \ll Z). \tag{41}$$

Therefore, from (39) - (41), we obtain the steady state value of D_Z as follows:

$$D_Z = \frac{K_7 D_{ZT}}{K_8 R_{ZT}} Z_T, \tag{42}$$

which is proportional to Z_T. Here, the assumptions $D_Z \ll K_7, K_8 \ll Z, Z \ll \frac{R_{ZT} K_8}{K_7}, R_{ZT} \ll Z$ should be satisfied, and these can be easily achieved by selecting a repressor based transcriptional sensor that has wide linear range.

Letting $dC_{RZ}/dt = 0$ in (37a), the steady state value of the complex, C_{RZ}, can be obtained as follows:

$$C_{RZ} = \frac{m_{RZ} R}{K_{RZ}}, \tag{43}$$

where $K_{RZ} = (b_{RZ} + k_{RZ} + \gamma)/a_{RZ}$ is the Michaelis-Menten constant. With (37a), (37b) and (43), and letting $m_{RZT} = m_{RZ} + C_{RZ}$ be the total concentration of the third repressor mRNA, we obtain follows:

$$m_{RZT} = \frac{d_{RZ} D_{RZ}}{\gamma} = m_{RZ} + C_{RZ} = m_{RZ}(1 + \frac{R}{K_{RZ}}) \rightarrow C_{RZ} = \frac{\frac{R}{K_{RZ}}}{1 + \frac{R}{K_{RZ}}} \frac{d_{RZ} D_{RZ}}{\gamma} \approx \frac{d_{RZ} D_{RZ}}{\gamma} \quad (\text{if } K_{RZ} \ll R). \tag{44}$$

From (37f) - (37g), letting $m_{ZT} = m_Z + C_9$ be the total concentration of the third mRNA, we obtain follows:

$$m_{ZT} = \frac{k_7 D_Z}{\gamma} = m_Z + C_9 = m_Z(1 + \frac{R}{K_9}) \rightarrow m_Z = \frac{k_7 D_Z}{\gamma(1 + \frac{R}{K_9})} \approx \frac{k_7 D_Z}{\gamma} \quad (\text{if } R \ll K_9). \tag{45}$$

By combining (38), (42) and (45), we obtain follows:

$$C_9 = \frac{\gamma k_7 K_7 D_{ZT}}{k_{RZ} d_{RZ} K_8 K_9 D_{RZ}} R Z_T, \tag{46}$$

which is proportional to R and Z_T. Here, the assumption $K_{RZ} \ll R \ll K_9$ should be satisfied and we can increase K_9 by decreasing TIR of the protease P_Z and decrease K_{RX} by increasing TIR of the repressor R_X. Also, from (37h), the steady state value of P_Z is equal to $k_9 C_9/\gamma$ and combining with (46), we obtain follows:

$$P_Z = \frac{k_7 k_9 K_7 D_{ZT}}{k_{RZ} d_{RZ} K_8 K_9 D_{RZ}} R Z_T \tag{47}$$

Here, conservation law of the resource R_{tot} in (33) should be modified as follow:

$$R_{tot} = R + C_3 + C_6 + C_9. \tag{48}$$

Then the steady state value of R can be derived as follow:

$$R = \frac{R_{tot}}{1 + c_1 X_T + c_2 Y_T + c_3 Z_T}, \tag{49}$$

where $c_3 = (\gamma k_7 K_7 D_{ZT})/(k_{RZ} d_{RZ} K_8 K_9 D_{RZ})$. Comparing to (33), the steady state value of R is decreased. However, according to (32), the steady state value of the output protein P_Y of Figure 3a is not a function of R and the only way that the additional resource competitor module can affect on the steady state value of the output protein P_Y is by changing the resource level R. Therefore, theoretically speaking, the steady state value of the output protein P_Y of Figure 3a and Figure 5a - incoherent merger network should be the same. On the other hand, according to (34), the steady state value of the output protein P_Y of Figure 4a is a function of R. Therefore, the steady state value of the output protein P_Y of Figure 5a - broken merging is smaller than that of Figure 4a because the resource level is decreased in (49).

Supplementary Note 2 Experimental Work

Supplementary Note 2.1 Genetic constructs in the main text

construct	RBS of *sfGFP* gene	TIR of *sfGFP* gene	protease tag of *sfGFP* gene	RBS of *mflon* gene	TIR of *mflon* gene	resource competitor module
Figure 3(a)	rbs1	8875	pdt#3	lib2130	903	n.a.
Figure 3(g)	lib1080	1770	pdt#3			n.a.
Figure 3(g)	lib1052	4575	pdt#3			n.a.
Figure 4(a)	rbs1	8875	n.a.			n.a.
Figure 5(b)	rbs1	8875	pdt#3			TetR-regulated RFP cassette
Figure 5(c)	rbs1	8875	n.a.			TetR-regulated RFP cassette

Supplementary Table 1: **List of all constructs used in this work.** The strength of ribosome binding sites (RBSs) in terms of translation initial rate (TIR) is predicted by the RBS calculator 2.0 [63] and thus, we tuned the GFP expression by using different RBSs. We selected the strongest protease tag, pdt#3, to efficiently degrade GFP proteins by the protease *mf*-Lon [64]. We implemented a TetR-regulated RFP cassette to serve as the resource competitor for free ribosomes [23]. n.a. stands for ”not available”. The plasmid maps are in Supplementary Figure 2 and Supplementary Figure 3 and full DNA sequences in GenBank files are provided in Supplementary Data.

Parameter	Unit	Value	Parameter	Unit	Value	Parameter	Unit	Value	Parameter	Unit	Value
a_{RX}	$\mu M^{-1}hr^{-1}$	21	b_{RX}	hr^{-1}	1	k_{RX}	hr^{-1}	2	d_{RX}	hr^{-1}	0.021
a_1	$\mu M^{-1}hr^{-1}$	174.6	b_1	hr^{-1}	1	k_1	hr^{-1}	1	a_2	$\mu M^{-1}hr^{-1}$	0.22
b_2	hr^{-1}	1	a_3	$\mu M^{-1}hr^{-1}$	1.31	b_3	hr^{-1}	1	k_3	hr^{-1}	12
a_{AY}	$\mu M^{-1}hr^{-1}$	21	b_{AY}	hr^{-1}	1	k_{AY}	hr^{-1}	1	d_{AY}	hr^{-1}	0.5
a_4	$\mu M^{-1}hr^{-1}$	0.0022	b_4	hr^{-1}	1	a_5	$\mu M^{-1}hr^{-1}$	0.0228	b_5	hr^{-1}	1
k_5	hr^{-1}	0.04	a_6	$\mu M^{-1}hr^{-1}$	0.1125	b_6	hr^{-1}	1	k_6	hr^{-1}	0.025
D_{RX}	μM	1	D_{XT}	μM	0.0126	D_{AY}	μM	1	D_{YT}	μM	5
γ	hr^{-1}	0.1 or 0.25	a	$\mu M^{-1}hr^{-1}$	100	b	hr^{-1}	1	k	hr^{-1}	1
X_T	μM	50 to 400	Y_T	μM	0 to 50	nominal R	μM	1	Z_T	nM	0 or 40
a_{RZ}	$\mu M^{-1}hr^{-1}$	15	b_{RZ}	hr^{-1}	1.5	k_{RZ}	hr^{-1}	1	d_{RZ}	hr^{-1}	0.045
a_7	$\mu M^{-1}hr^{-1}$	150.3	b_7	hr^{-1}	1	k_7	hr^{-1}	1	a_8	$\mu M^{-1}hr^{-1}$	0.3
b_8	hr^{-1}	1	a_9	$\mu M^{-1}hr^{-1}$	1	b_9	hr^{-1}	1	k_9	hr^{-1}	10

Supplementary Table 2: **Simulation parameters to generate the reduced models and the full models in Figure 2-5.** According to Supplementary Figure 8 - Supplementary Figure 15, growth rate of the broken merging constructs is much faster than that of the incoherent merger networks, so we use larger γ (dilution rate or growth rate) for the broken merging constructs.

Supplementary Note 2.2 Additional experimental data

```
pRSA  AGCTGTCACCGGATGTGCTTTCCGGTCTGATGAGTCCGTGAGGACGAAACAGCCTCTACAAATAATTTTGTTTAAACTAGAAGGAGGAAAAAAATG
pRSB  AGCTGTCACCGGATGTGCTTTCCGGTCTGATGAGTCCGTGAGGACGAAACAGCCTCTACAAATAATTTTGTTTAATGGTTTCGTAAATAAGGAACTTTATATG
pRSC  AGCTGTCACCGGATGTGCTTTCCGGTCTGATGAGTCCGTGAGGACGAAACAGCCTCTACAAATAATTTTGTTTAATGGTTTTTTAAATAAGGAACTTTATATG
```

Supplementary Figure 1: **Alignment of 5'-UTR of the superfolder GFP (*sfGFP*) gene of the constructs in Figure 3.** RiboJ ribozyme and its cleavage site [65] are highlighted in yellow and magenta colors, respectively. P_{salTTC} promoter and ribozyme (RiboJ) of *sfGFP* gene are cloned without any modification from pAJM.771 plasmid [34]. Ribosome binding site (RBS) are the bases in bold font and red color and are designed by the RBS calculator 2.0 [63]; they are rbs1, lib1080, and lib1052 for pRSA (TIR = 8875), pRSB (TIR = 1770), and pRSC (TIR = 4575), respectively. The initial codon ATG of *sfGFP* gene is highlighted in green color. The predicted TIRs at the initial codon are listed respectively in Supplementary Table 1.

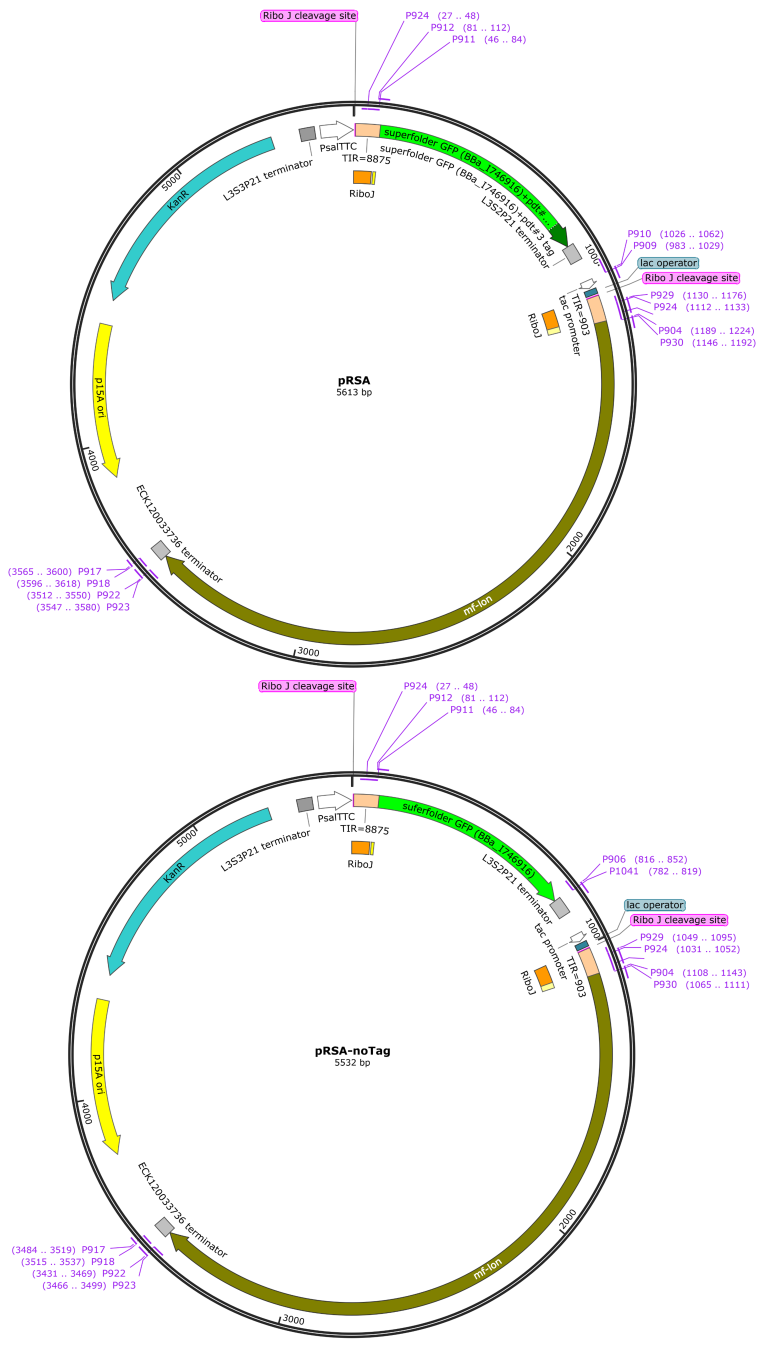

Supplementary Figure 2: **The plasmid maps of Figure 3a and Figure 4a constructs.** The primers used in Golden Gate assembly are the purple text labels. All essential features of the map are annotated. Figure 4a is removed the pdt#3 tag of superfolder GFP gene from Figure 3a. P_{tac} promoter with *lac* operator and ribozyme (RiboJ) of *mflon* gene are cloned without any modification from pAJM.336 plasmid [34].

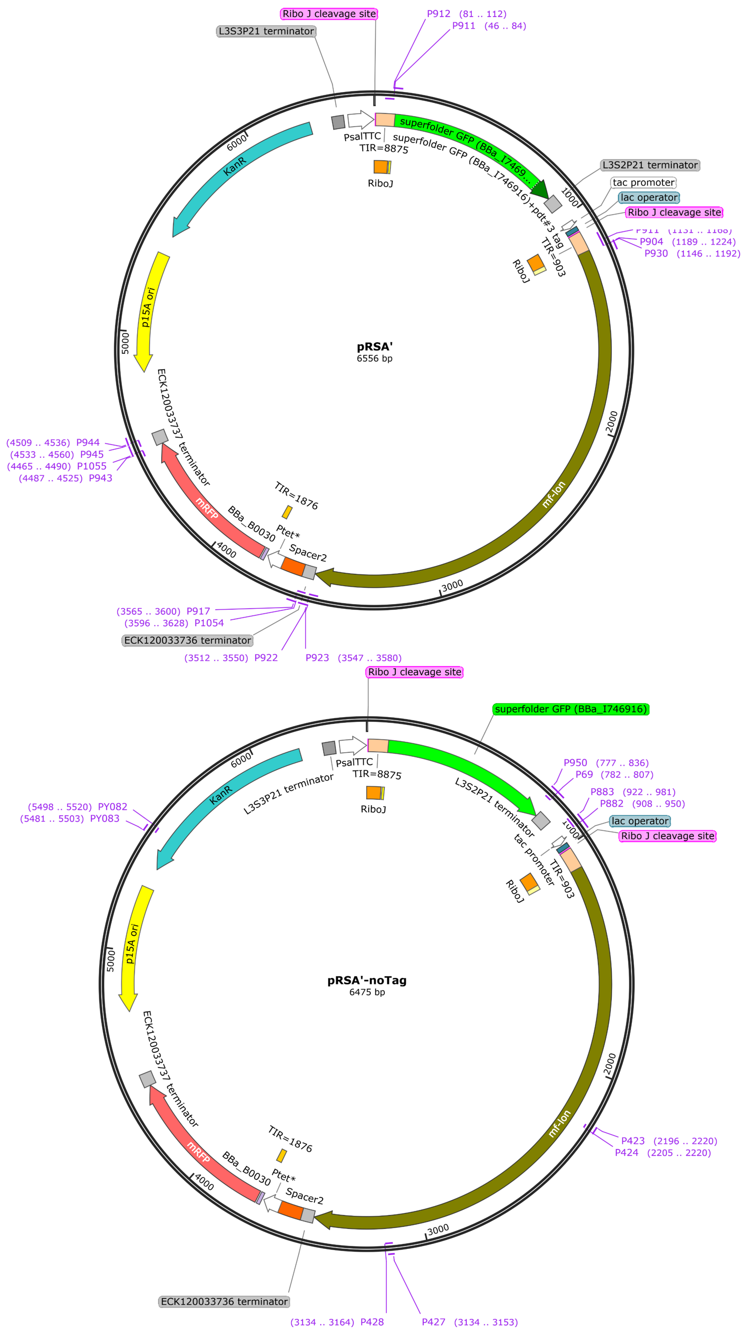

Supplementary Figure 3: **The plasmid maps of Figure 5a - incoherent merger network and Figure 5a - broken merging constructs.** The primers used in assembly are the purple text labels. Figure 5a - incoherent merger network and Figure 5a - broken merging use Golden Gate and Gibson assembly, respectively. All essential features of the map are annotated. Note that Figure 5a - incoherent merger network is implemented a TetR-regulated RFP expression cassette in downstream of the *mfLon* cassette, compared to Figure 3a. Figure 5a - broken merging is removed the pdt#3 tag of superfolder GFP gene from Figure 4a. P_{tac} promoter with *lac* operator and ribozyme (RiboJ) of *mflon* gene are cloned without any modification from pAJM.336 plasmid [34].

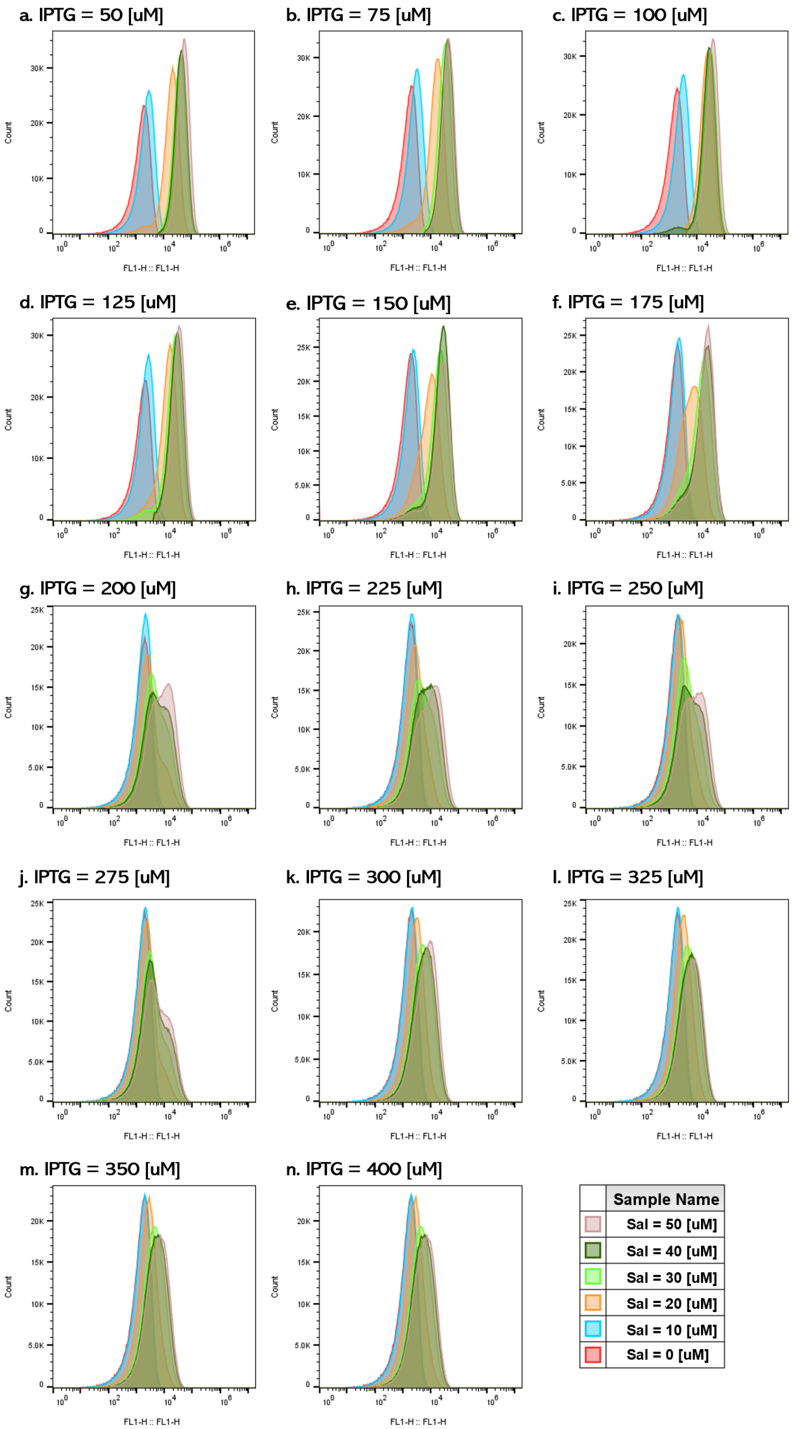

Supplementary Figure 4: **Flow cytometry data of Figure 3a.** Flow cytometry data of Figure 3a with fixed IPTG values and different Sal values. One biological replicate is measured by Accuri C6 flow cytometer (Becton Dickinson, Special Order 2B2LYG RUO System, 656035) to generate histogram of FL1-H versus count. The detection threshold was set as 7000 on FSC-H channel. The singlet events are at least 70000 counts and analyzed by the FlowJo v10 (FlowJo, LLC).

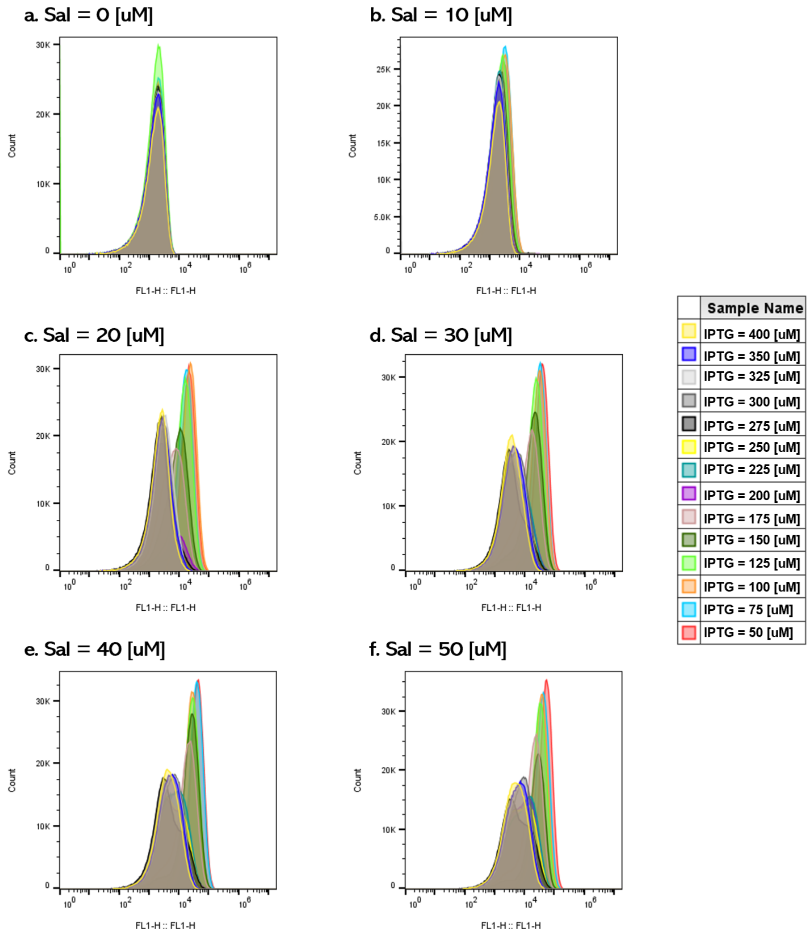

Supplementary Figure 5: **Flow cytometry data for of Figure 3a.** Flow cytometry data of Figure 3a with fixed Sal values and different IPTG values. One biological replicate is measured by Accuri C6 flow cytometer (Becton Dickinson, Special Order 2B2LYG RUO System, 656035) to generate histogram of FL1-H versus count. The detection threshold was set as 7000 on FSC-H channel. The singlet events are at least 70000 counts and analyzed by the FlowJo v10 (FlowJo, LLC).

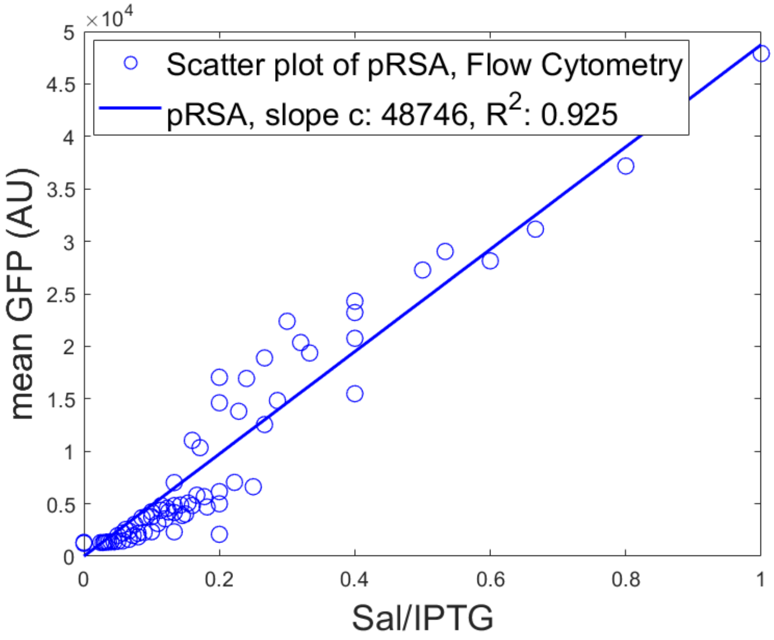

Supplementary Figure 6: **Scatter plot of mean GFP verses Sal/IPTG of Figure 3a obtained from Flow cytometry data.** We use geometric mean of GFP value from histogram of Supplementary Figure 4, which is equivalent to Supplementary Figure 5, with proper Sal and IPTG concentrations to plot the scatter plot.

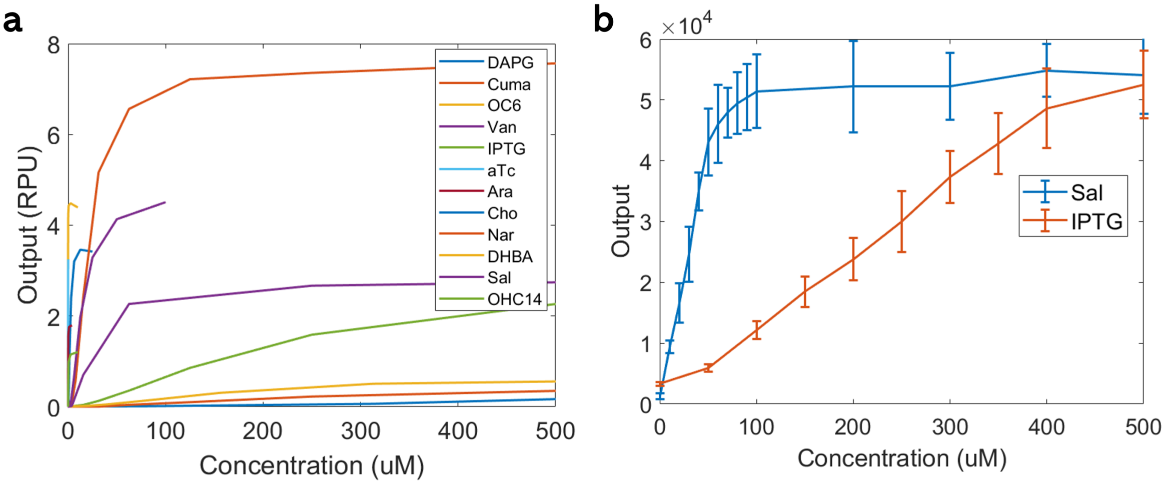

c

	Linear Range[uM]	Comments
**DAPG**	0~10	Low Range
**Cuma**	0~100	Solvent: Ethanol
**OC6**	0~0.1	Low Range
**Van**	0~10	Solvent: Ethanol, Low Range
**IPTG**	50~400	Solvent: Water
**aTc**	0~0.04	Low Range
**Ara**	0~100	Requires additional gene to use
**Cho**	N/A	Low Range
**Nar**	N/A	Solvent: DMSO, Low Range
**DHBA**	0~400	Solvent: Ethanol
**Sal**	0~50	Solvent: Water
**OC14**	0~0.05	Low Range

Supplementary Figure 7: **Dose response curves of transcriptional sensors in [34].** Upper left shows dose response curve of twelve transcriptional sensors obtained from the first author of [34]. Upper right shows dose response curve of IPTG and Sal transcriptional sensors that we obtain. Data are presented as mean values ± SD of n=3 biologically independent experiments. Bottom shows the reason that we choose IPTG and Sal transcriptional sensors among others.

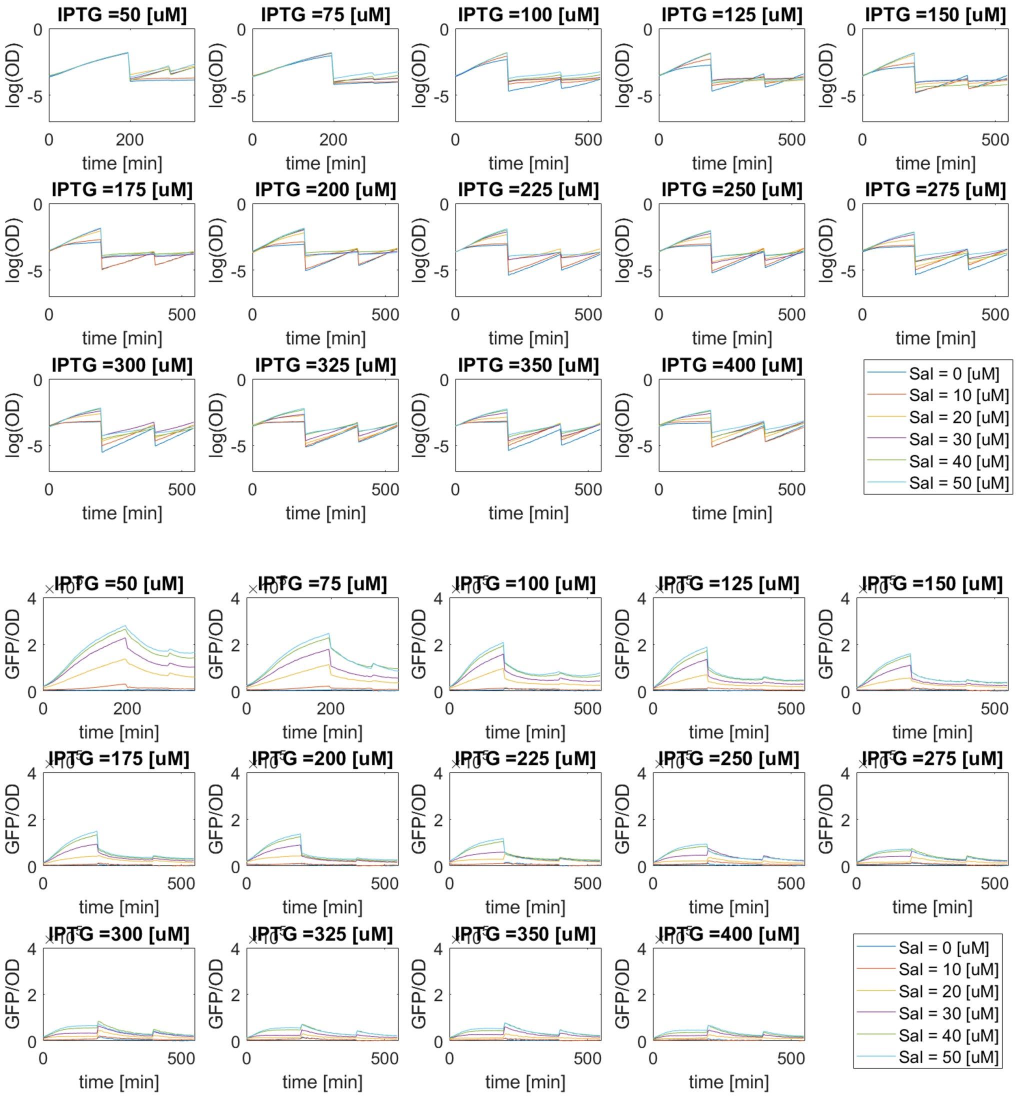

Supplementary Figure 8: **Exponential growth data and GFP/OD data of Figure 3a.** Each trace represents the growth curve and GFP expression of different IPTG and Sal combination. To analyze GFP/OD values, we choose the data point at the second batch with an OD value most close to 0.054 for each experiment condition.

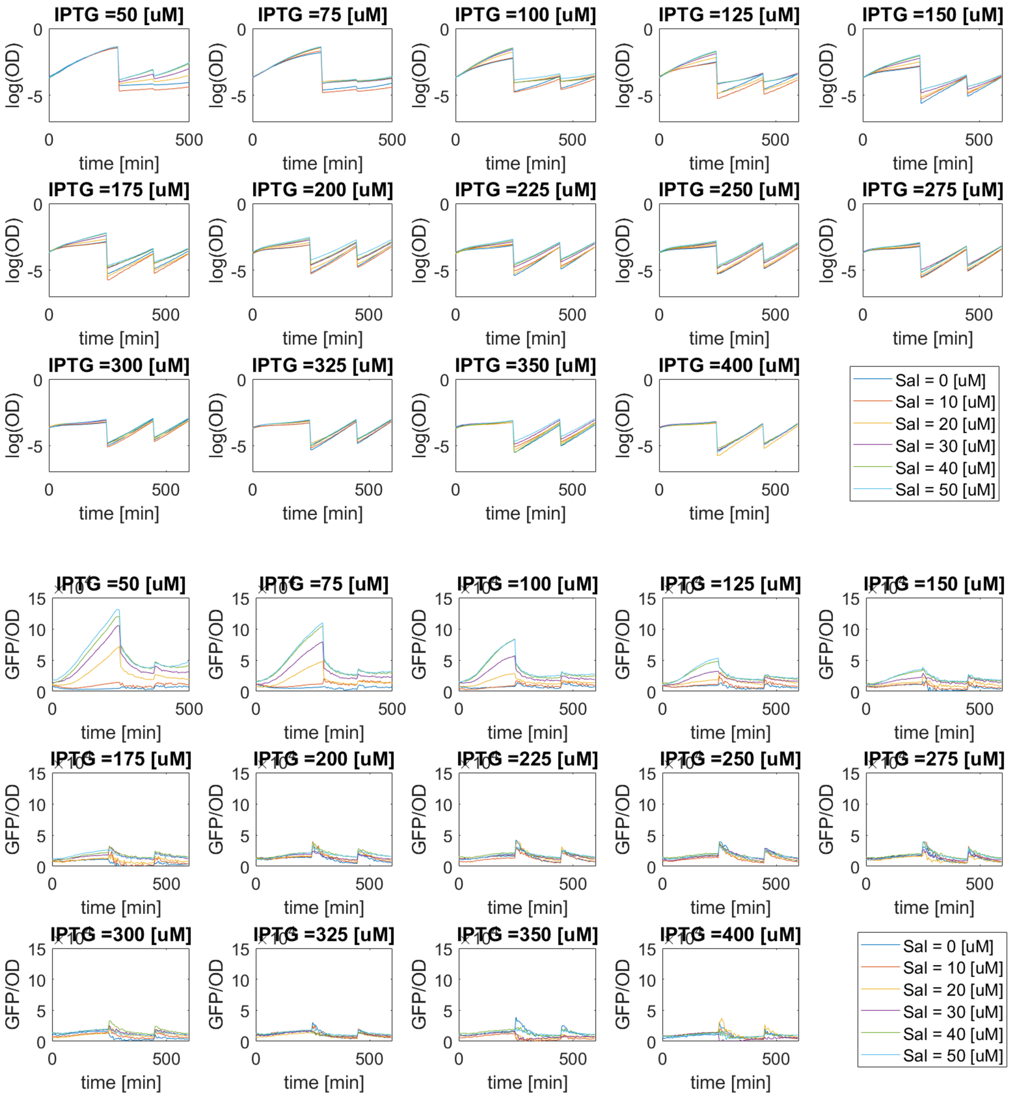

Supplementary Figure 9: **Exponential growth data and GFP/OD data for pRSB (TIR = 1770) in Figure 3a.** Each trace represents the growth curve and GFP expression of different IPTG and Sal combination. To analyze GFP/OD values, we choose the data point at the second batch with an OD value most close to 0.054 for each experiment condition.

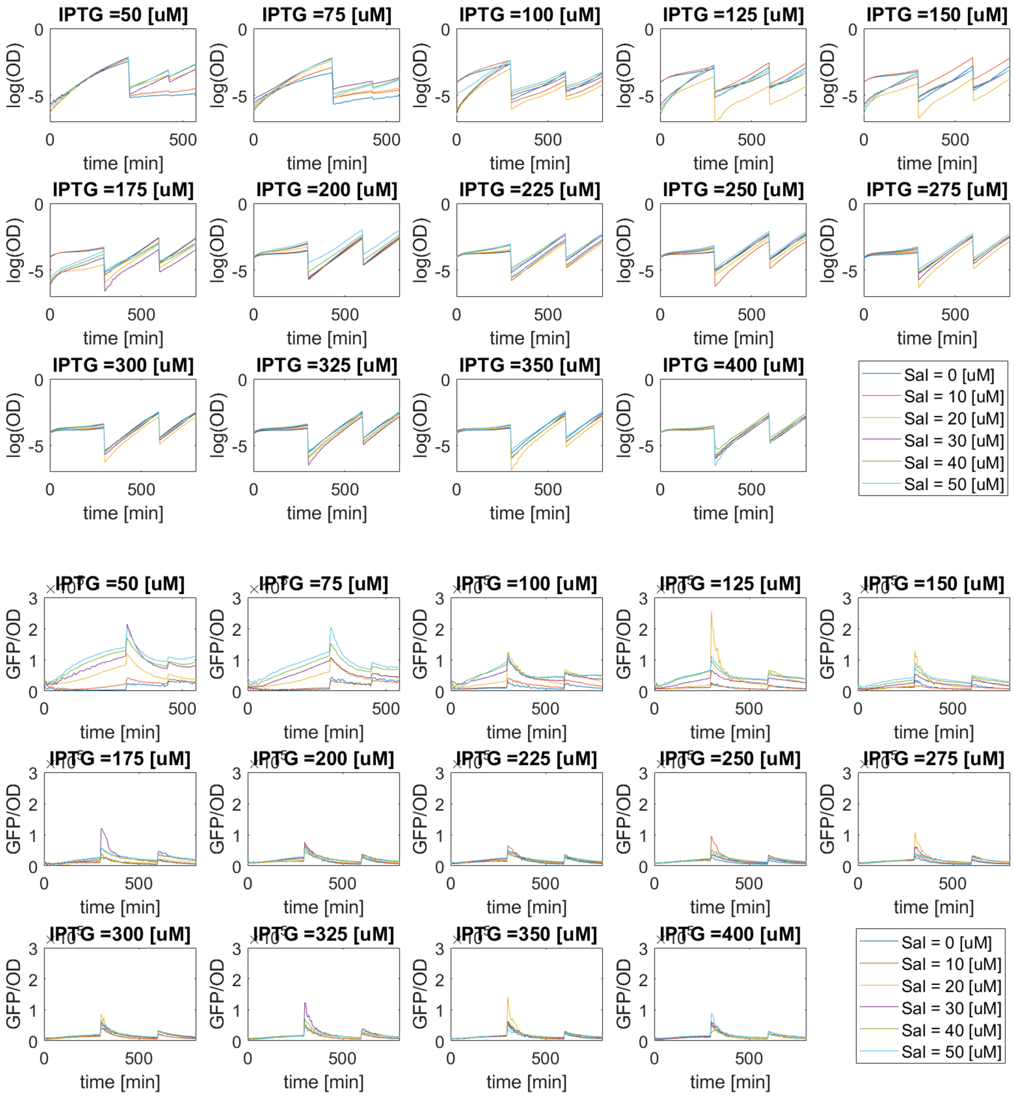

Supplementary Figure 10: **Exponential growth data and GFP/OD data for pRSC (TIR = 4575) in Figure 3a.** Each trace represents the growth curve and GFP expression of different IPTG and Sal combination. To analyze GFP/OD values, we choose the data point at the second batch with an OD value most close to 0.054 for each experiment condition.

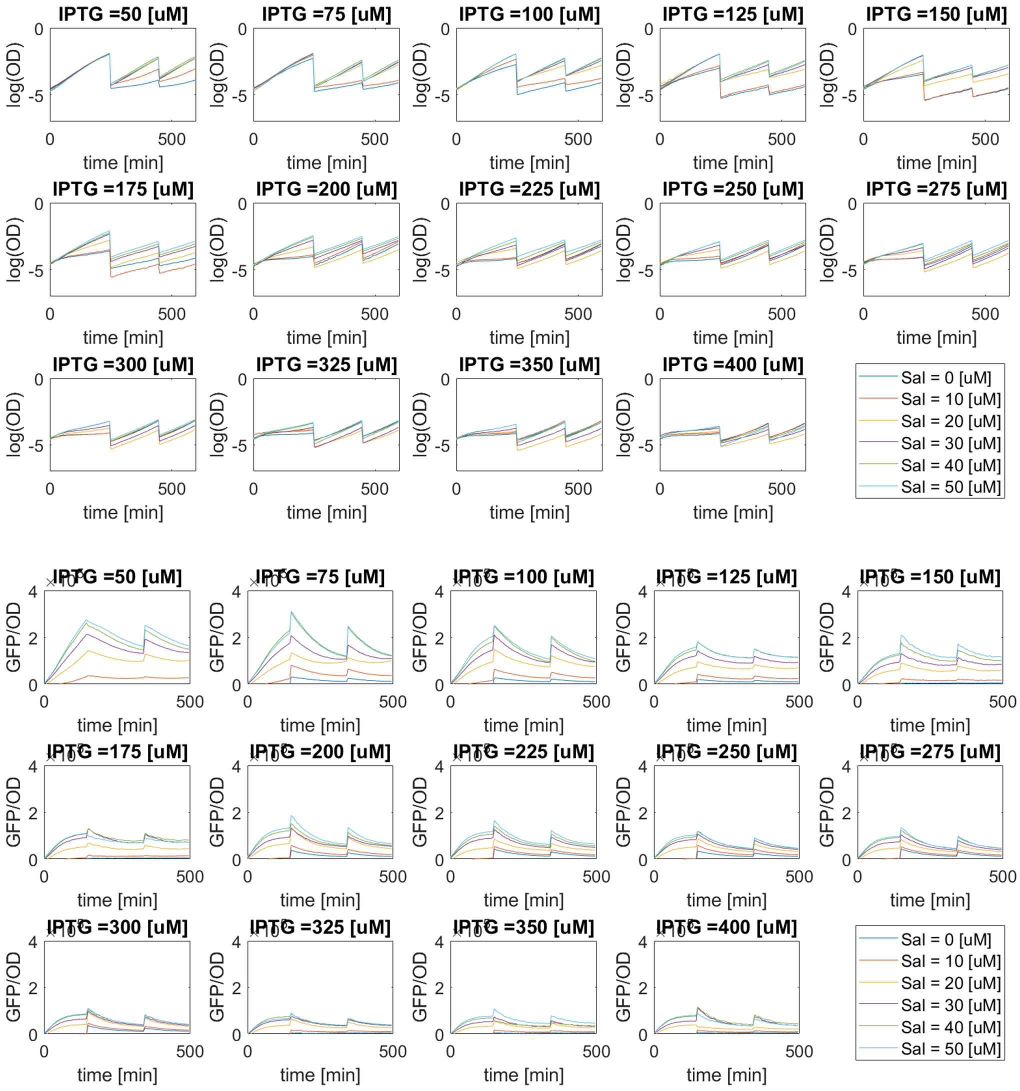

Supplementary Figure 11: **Exponential growth data and GFP/OD data of Figure 4a.** Each trace represents the growth curve and GFP expression of different IPTG and Sal combination. To analyze GFP/OD values, we choose the data point at the second batch with an OD value most close to 0.134 for each experiment condition.

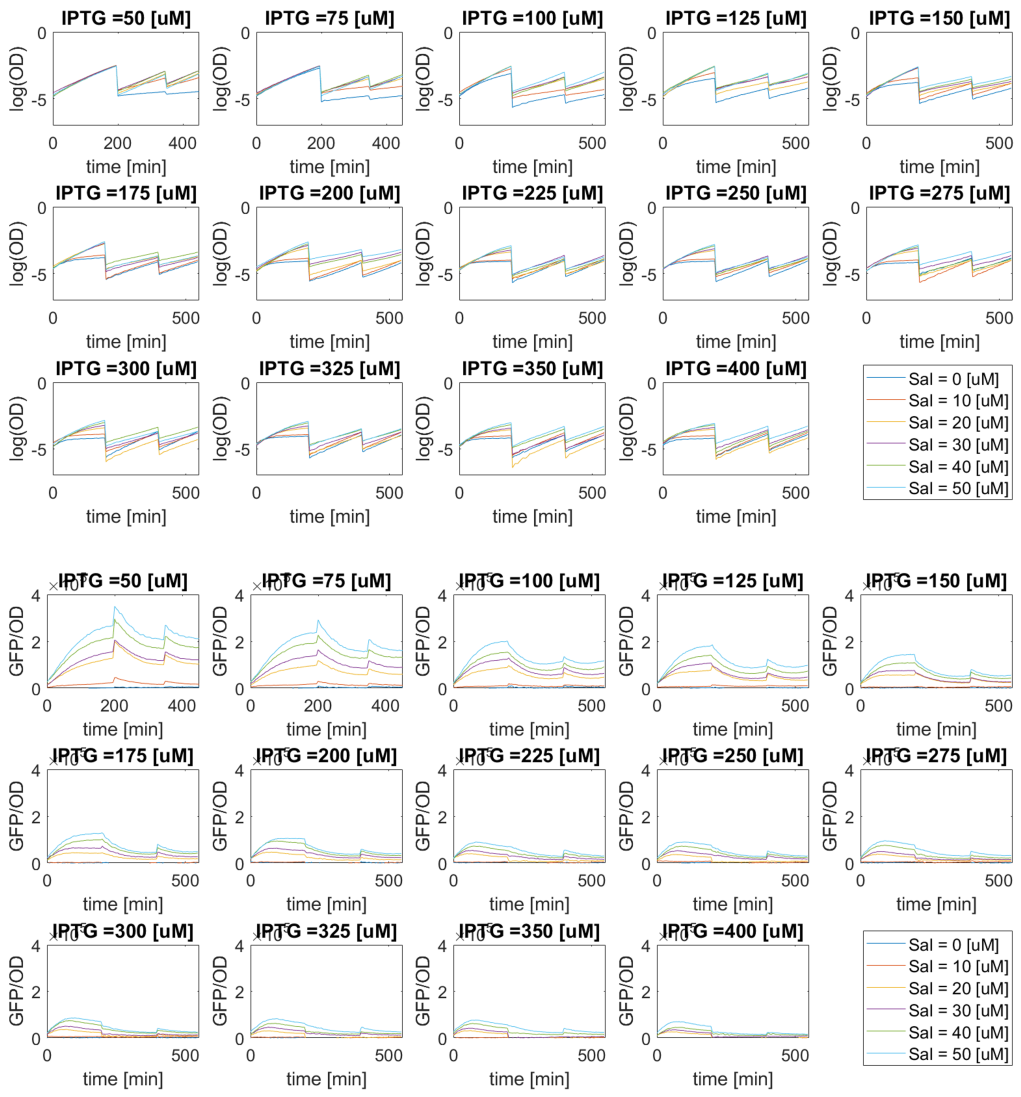

Supplementary Figure 12: **Exponential growth data and GFP/OD data of Figure 5a - incoherent merger network with aTc = 0 [nM].** Each trace represents the growth curve and GFP expression of different IPTG and Sal combination. To analyze GFP/OD values, we choose the data point at the second batch with an OD value most close to 0.054 for each experiment condition.

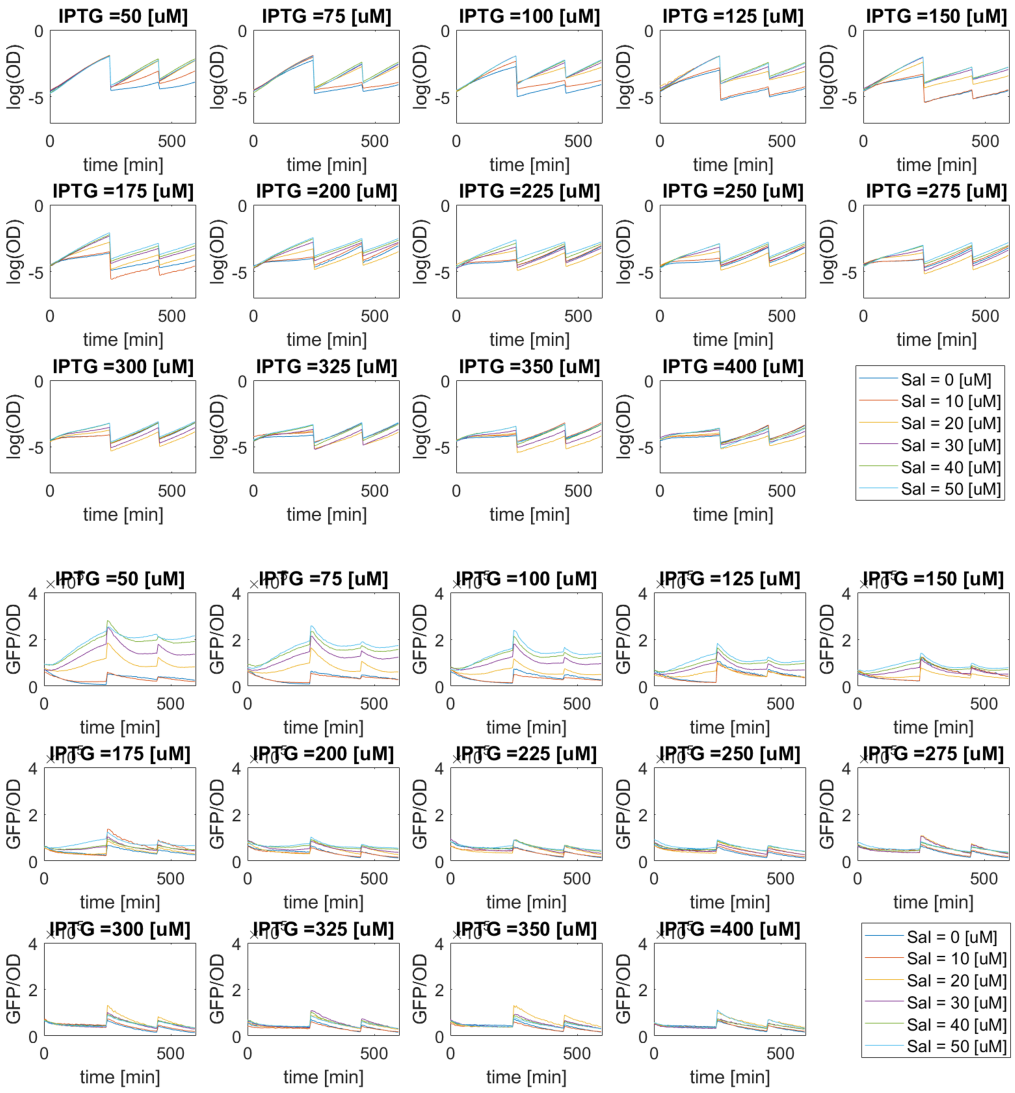

Supplementary Figure 13: **Exponential growth data and GFP/OD data of Figure 5a - incoherent merger network with aTc = 40 [nM].** Each trace represents the growth curve and GFP expression of different IPTG and Sal combination. To analyze GFP/OD values, we choose the data point at the second batch with an OD value most close to 0.054 for each experiment condition.

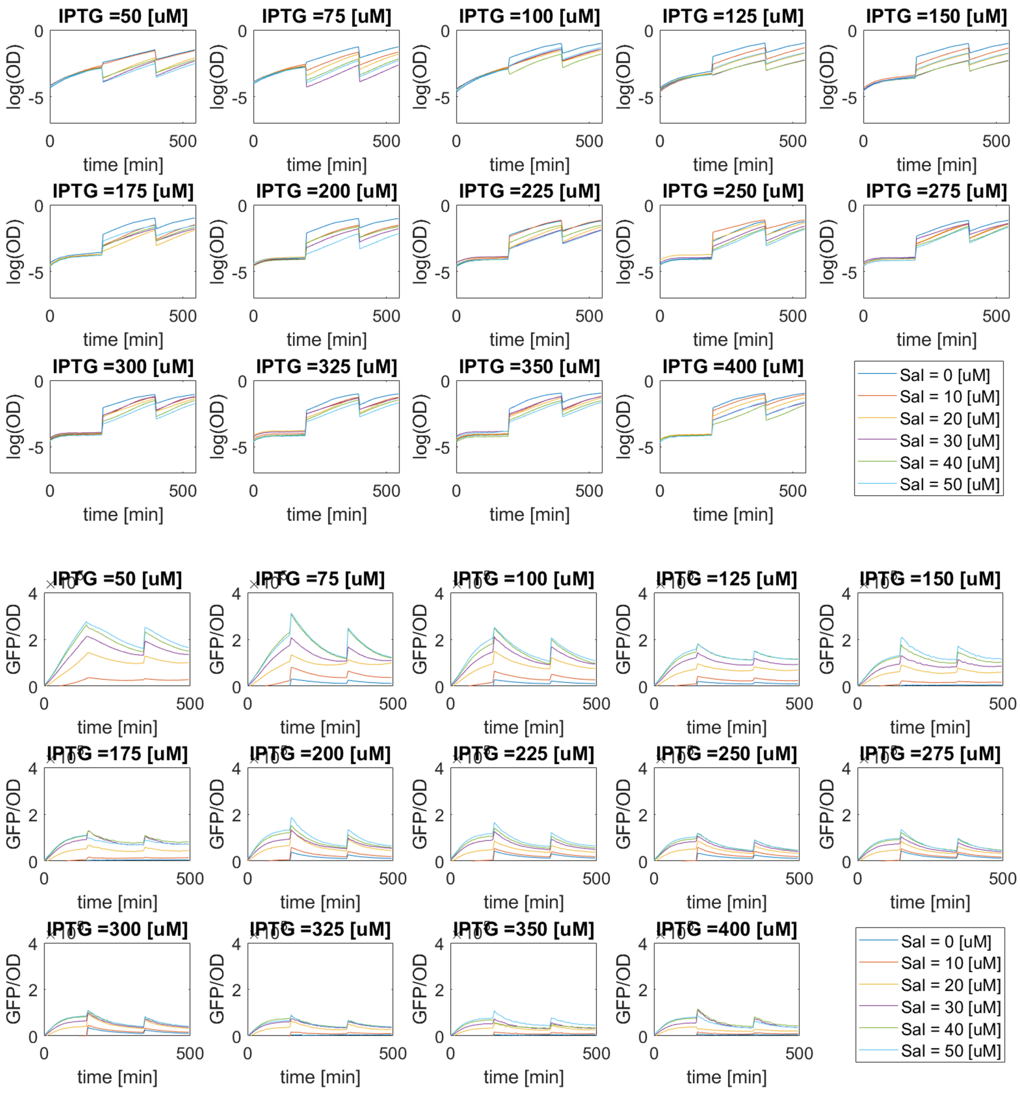

Supplementary Figure 14: **Exponential growth data and GFP/OD data of Figure 5a - broken merging with aTc = 0 [nM].** Each trace represents the growth curve and GFP expression of different IPTG and Sal combination. To analyze GFP/OD values, we choose the data point at the second batch with an OD value most close to 0.134 for each experiment condition.

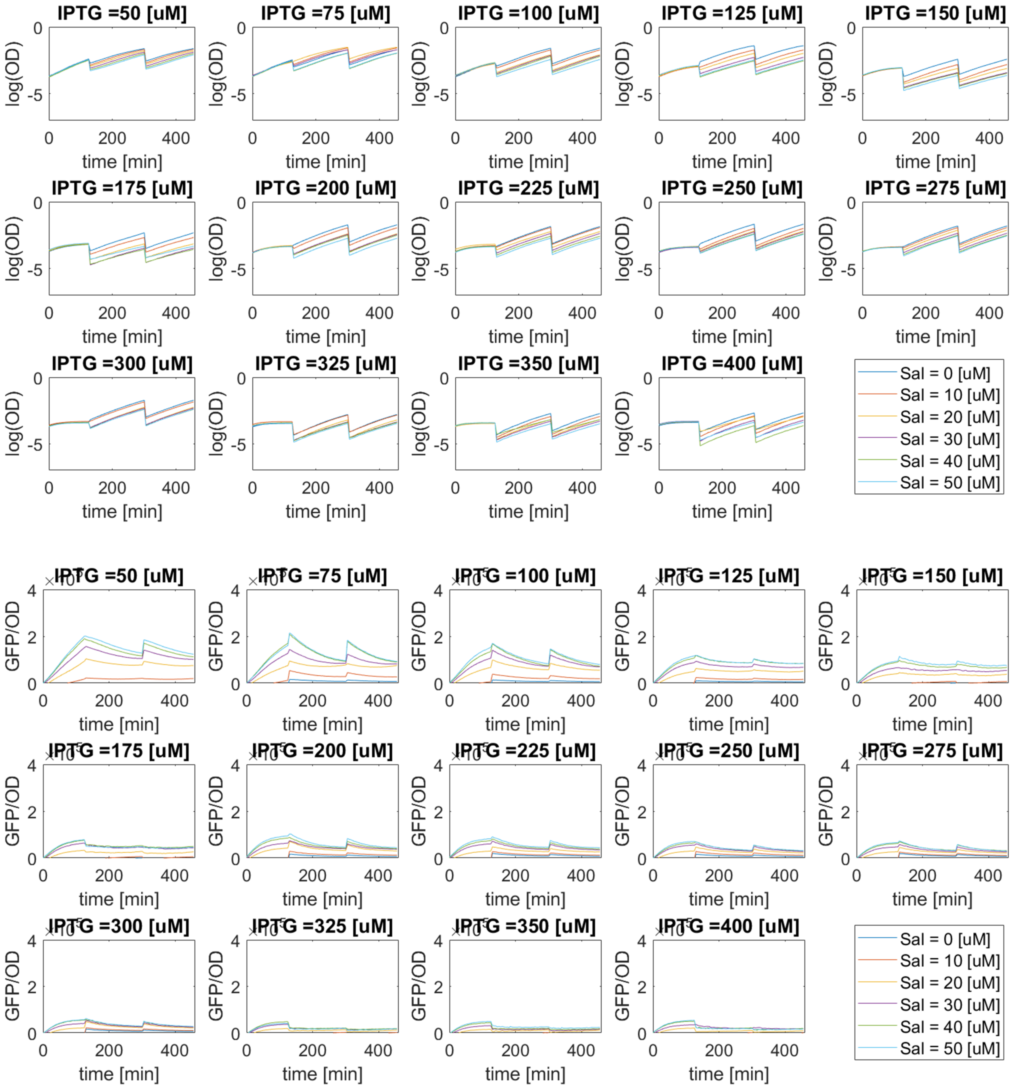

Supplementary Figure 15: **Exponential growth data and GFP/OD data of Figure 5a - broken merging with aTc = 40 [nM].** Each trace represents the growth curve and GFP expression of different IPTG and Sal combination. To analyze GFP/OD values, we choose the data point at the second batch with an OD value most close to 0.134 for each experiment condition.
